# Supplementary material for: Mixed Matrix Membranes for Efficient CO2 Separation Using an Engineered UiO-66 MOF in a Pebax Polymer
Source: Polymers (Basel). 2022 Feb 9;14(4):655. doi: 10.3390/polym14040655 (PMC8880452; doi:10.3390/polym14040655)
Supplement: Supplementary file 1 [file polymers-14-00655-s001.zip › polymers-1568309-supplementary.pdf]

Supporting Information for

Mixed Matrix Membranes for Efficient CO_2 Separation Using an Engineered UiO-66 MOF in a Pebax Polymer

Asmaul Husna [1,2], Iqubal Hossain [1,2,*], Insu Jeong [1,2] and Tae-Hyun Kim [2,*]

[1] Organic Material Synthesis Laboratory, Department of Chemistry, Incheon National University, Incheon 22012, Korea
[2] Research Institute of Basic Sciences, Incheon National University, Incheon 22012, Korea

* iqubal.chem.ru.08@gmail.com (I.H.); tkim@inu.ac.kr (T.-H.K.)

This file includes:

Characterization and measurement

Scheme S1

Figures S1–S8

Tables S1–S4

S1. Characterization and Measurements

^{1}H NMR spectra were collected with an Agilent 400-MR (400 MHz) instrument using $CDCl_3$ and D_2O as the solvents and TMS as a reference or internal deuterium lock. ATR-FTIR spectra were recorded on a PerkinElmer FTIR Spectrum Two.

Membrane thickness was determined with a micrometer (Mitutoyo Model 547-201, Japan).

Thermogravimetric analysis (TGA) was conducted on a Scinco TGA/N-1000 analyzer at a heating rate of 10 °C min^{-1} under a nitrogen flow to determine the thermal properties of the samples.

Wide-angle X-ray diffraction (WAXD) spectra of the dry membranes were collected using a Rigaku HR-XRD Smart Lab diffractometer at a scanning rate of 0.2° min^{-1} in a 2θ range from 5° to 50° with a Cu-Kα X-ray source (λ = 1.54 Å). The dried membranes were placed under vacuum at 80 °C for 12 h and were equilibrated at 50% RH at least 24 h prior to the measurement.

Field emission scanning electron microscopy (FE-SEM) was performed to investigate the surface and cross-sectional morphologies of the membranes and the distribution and size of the fillers embedded into the polymer matrix. Moreover, to obtain the cross-sectional morphology, the membranes were first fractured in liquid nitrogen and then coated with gold. All membranes have a thickness between 50 and 60 mm.

Atomic force microscopy (AFM) was performed using a Bruker Multi-Mode instrument in a Tapping mode. A silicone cantilever with an end radius < 10 nm and a force constant of 40 N m^{-1} (NCHR, nanosensors, f = 300 kHz) was used to image the samples at ambient temperature. The samples were equilibrated with 50% RH at least 24 prior to the imaging. The measurements were conducted under the same conditions for each sample to keep consistency.

Nitrogen adsorption experiments were conducted at 77 K using a Micromeritics ASAP 2020 HD88. All the samples were degassed under a vacuum at 150 °C for 10 h prior to the analysis. The surface areas were calculated from a multipoint BET plot. Nitrogen sorption isotherms were measured at 77 K. All samples were degassed at 150 °C for 2 h under a vacuum before being testing.

Tensile properties were measured on a Shimadzu EZ-TEST E2-L instrument benchtop tensile tester using a crosshead speed of 1 mm min^{-1} at 25 ºC under 50% relative humidity. The membranes have a thickness between 50 and 60 mm. Engineering stress was calculated from the initial cross sectional area of the sample and Young's modulus (E) was determined from the initial slope of the stress–strain curve. The membrane samples were cut into a rectangular shape with 40 mm × 10 mm (total) and 20 mm × 10 mm (test area)

S2. Gas Permeation Measurements

Permeation measurements of pure gases were taken using a high-vacuum time-lag measurement unit based on a constant-volume/variable-pressure method. All experiments were performed at a feed pressure of 1 bar (except for the plasticization experiments, which were carried out at 1–20 bar feed pressures) and a feed temperature of 30 °C. Before taking these measurements, to remove any residual gases, both the feed and the permeate sides were thoroughly evacuated to below 10^{-5} Torr (1.33×10^{-8} bar) until the readout showed zero. The downstream volume was calibrated using a Kapton membrane and was found to be 57 cm^3. The upstream and downstream pressures were measured using a Baraton transducer (MKS; Model No. 626B02TBE) with full scales of 10,000 and 2 Torr (13.3 and 2.7×10^{-3} bar), respectively. The pressure on the permeate side was recorded as a function of time using a pressure transducer and recorded by a desktop computer through a shielded data cable. The permeability coefficient was determined from the linear slope of the downstream pressure versus time plot (dp/dt) according to the following equation:

$$P = \frac{273}{76} \times \frac{V_1}{AT\rho_o} \times \frac{dp}{dt} \qquad (1)$$

where P is the permeability expressed in Barrer(1 Barrer = 10^{-10} [cm^3 (STP) cm cm^{-2} s^{-1} cm Hg^{-1}], V (cm^3) is the downstream volume, l (cm) is the membrane thickness, A (cm^2) is the effective area of the membrane, T (K) is the measurement temperature, p_o (Torr) is the pressure of the feed gas in the upstream chamber, and dp/dt is the rate of the pressure change at a steady state. For each gas, the permeation tests were repeated a minimum of three times, and the standard deviation from the mean values of the permeabilities was within ~3%. The sample-to-sample reproducibility was high and within 3%. The effective membrane areas were 15.9 cm^2.

The ideal perm-selectivity, $\alpha_{A/B}$, of the membrane for a pair of gases (A and B) is defined as the ratio of the individual gas permeability coefficients:

$$\alpha_{A/B} = \frac{P_A}{P_B} \qquad (2)$$

The diffusivity and solubility were obtained from the time-lag (θ) value according to the following equations:

$$D = \frac{l^2}{6\theta} \qquad (3)$$

$$S = \frac{P}{D} \qquad (4)$$

where D ($cm^2\ s^{-1}$) is the diffusivity coefficient, l (cm) is the membrane thickness, and θ (s) is the time lag, as obtained from the intercept of the linear steady-state part of the downstream pressure versus time plot. The solubility, S, was calculated from Equation (4) with the permeability and diffusivity obtained from Equations (1) and (3).

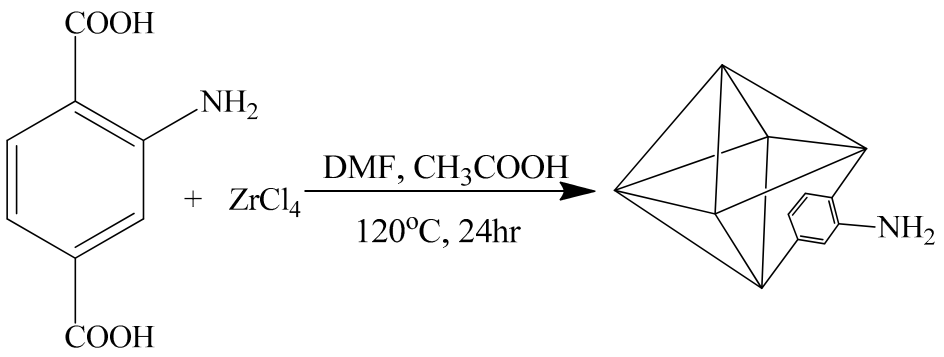

Scheme S1: Synthesis of UiO-66-NH_2

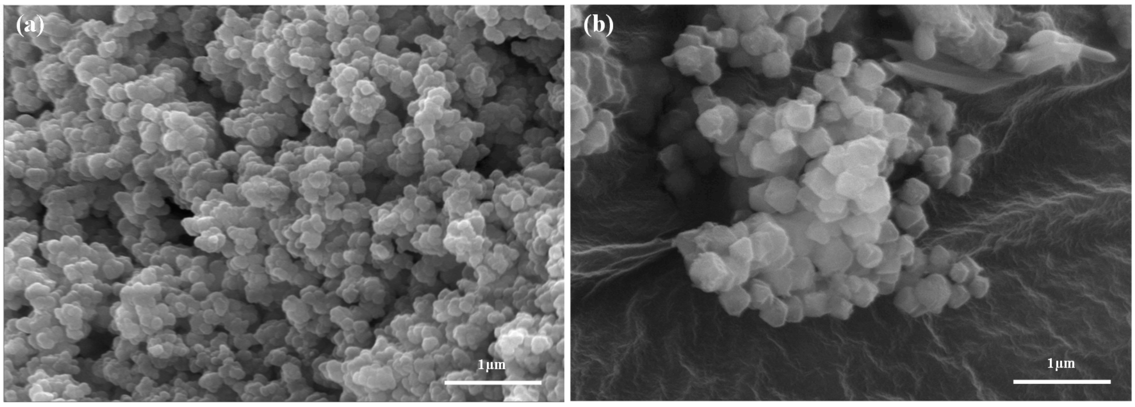

Figure S1. SEM images of (**a**) UiO-66-NH_2, and (**b**) PIM-grafted-MOF (PIM-g-MOF)

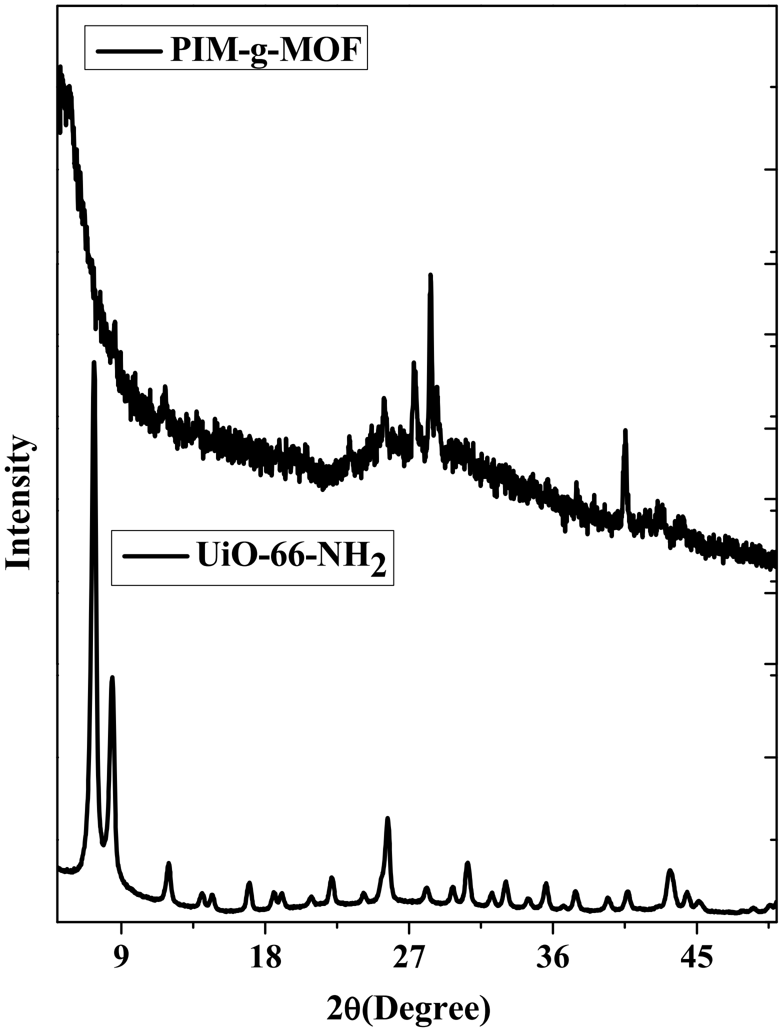

Figure S2. XRD spectra for UiO-66-NH_2 and PIM-g-MOF particles.

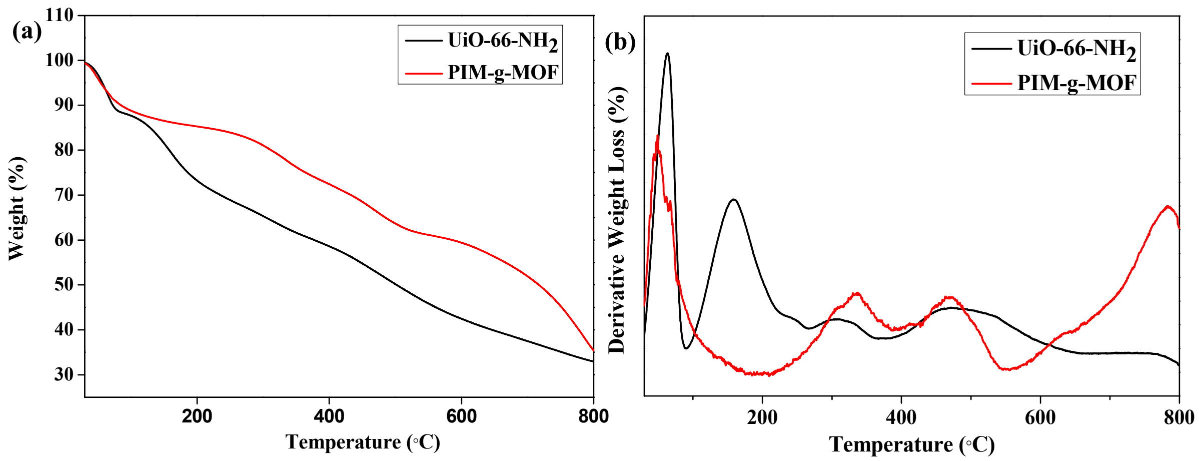

Figure S3. (**a**) TGA and (**b**) DTG curves for UiO-66-NH2 and PIM-g-MOF particles.

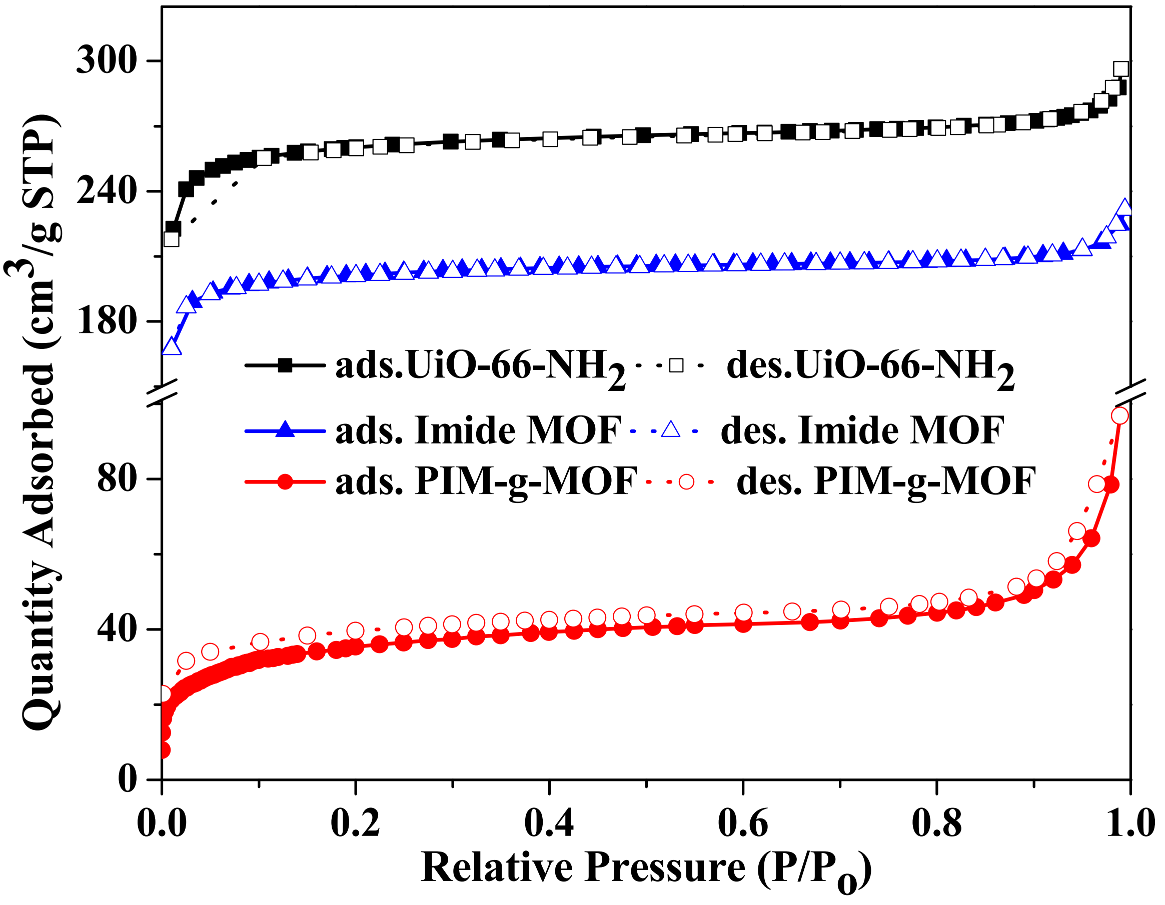

Figure S4. Nitrogen adsorption (solid line-filled symbol)/desorption (dotted line-empty symbol) isotherm BET plots at 77 K for UiO-66-NH2, imide MOF, and PIM-g-MOF.

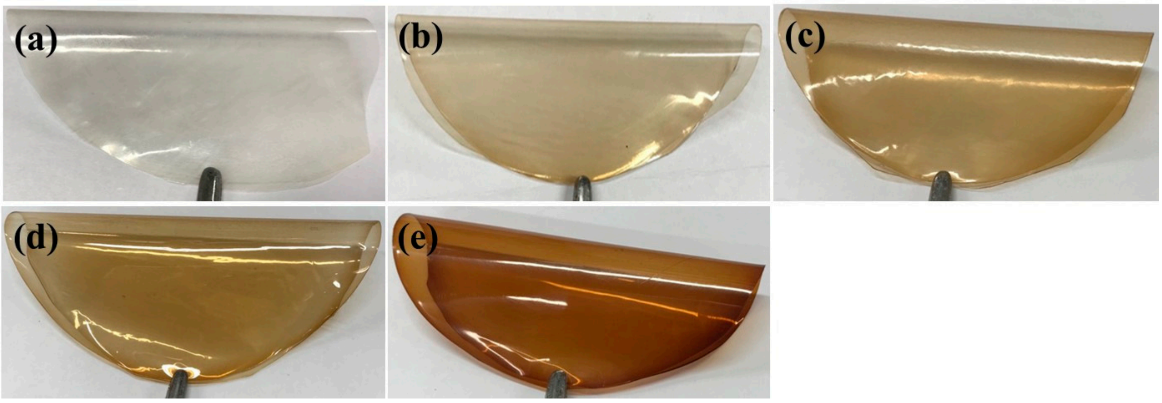

Figure S5. Photographs of (**a**) pristine Pebax membrane and MMMs with (**b**) 0.5 wt%, (**c**) 1 wt%, (**d**) 3 wt% and (**e**) 5 wt% of PIM-g-MOF loading.

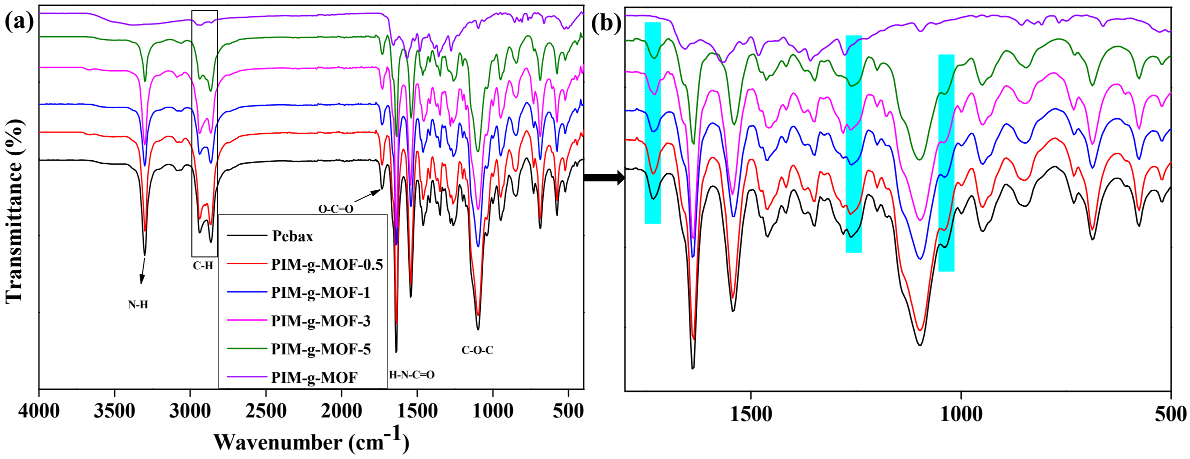

Figure S6. (**a**) ATR-FTIR spectra of the pristine Pebax membrane, MMMs with various MOF (PIM-g-MOF) loading, and (**b**) the same spectra from 1800 to 500 cm^{-1}.

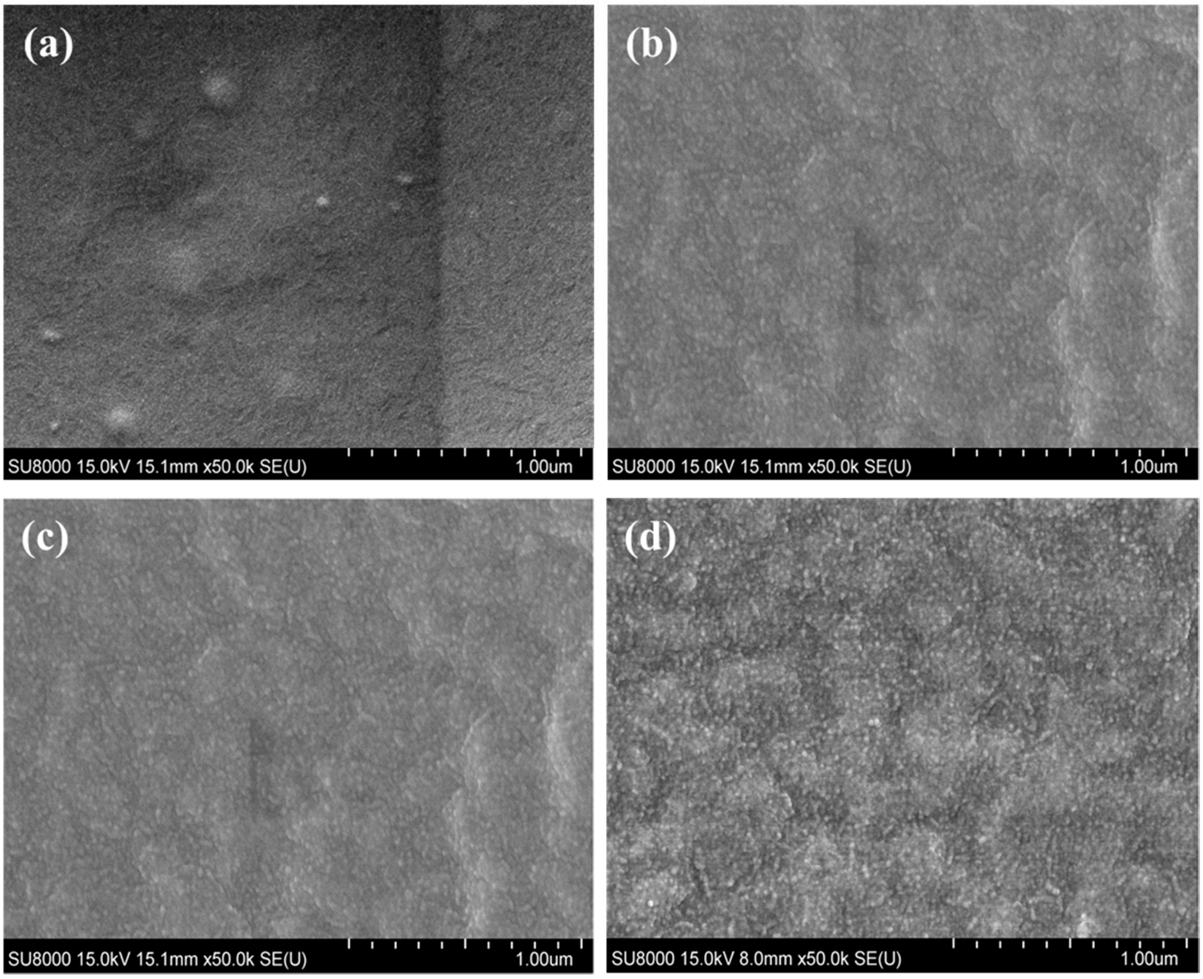

Figure S7. Cross-sectional SEM images of various loadings of fillers: (**a**) PIM-g-MOF-0.5, (**b**) PIM-g-MOF-1, (**c**) PIM-g-MOF-3, and (**d**) PIM-g-MOF-5.

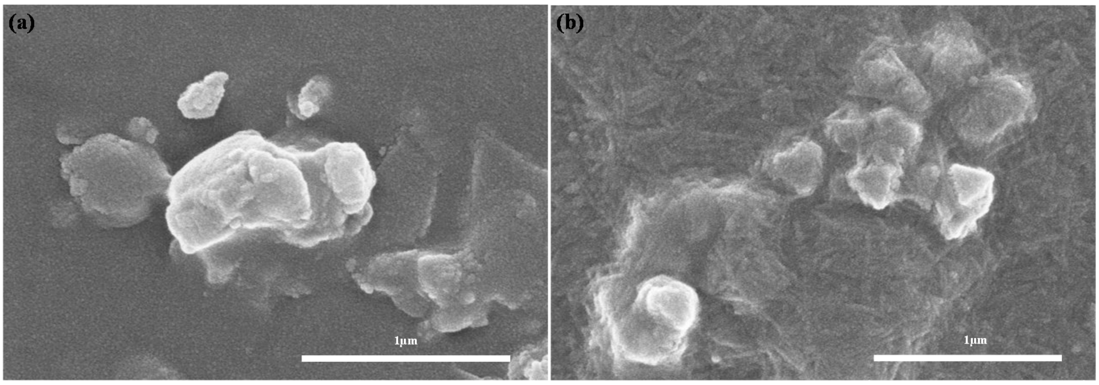

Figure S8. SEM images of (**a**) UiO-66-NH_2-1 and (**b**) UiO-66-NH_2-3.

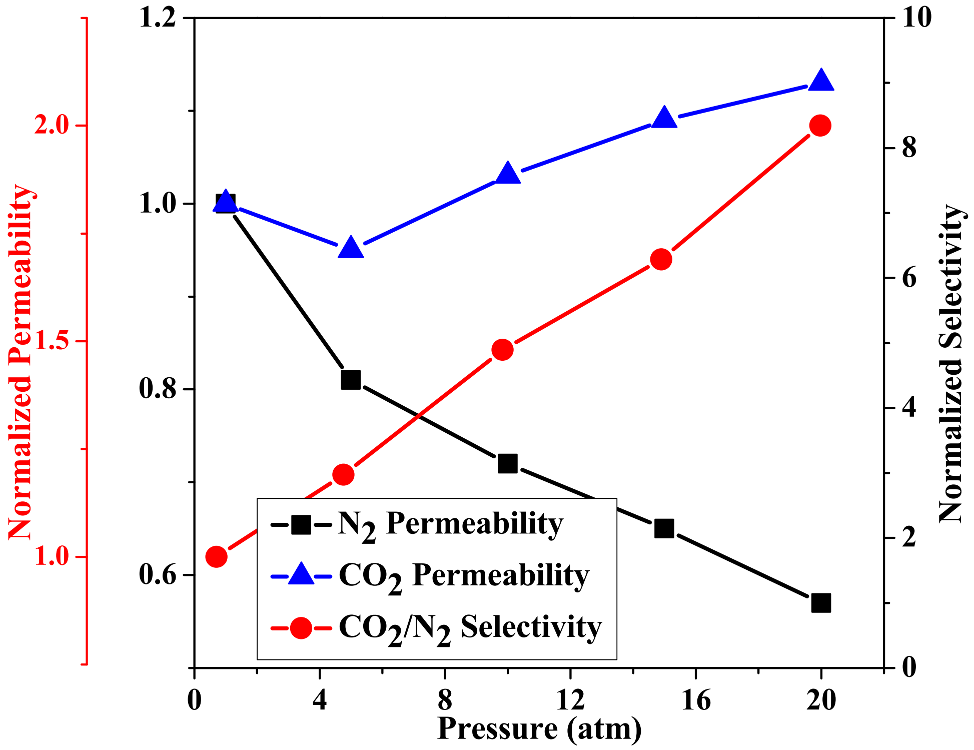

Figure S9. Pressure effect on the CO_2, N_2 permeability and CO_2/N_2 selectivities. Permeability is normalized by the initial permeability with feed pressure at 2atm.

Table S1. Thermal properties of the MMMs obtained from TGA analysis

Membrane	T_d (°C)	T_{max} (°C)
Pebax	350	416
PIM-g-MOF-0.5	348	418
PIM-g-MOF-1	346	417
PIM-g-MOF-3	341	414
PIM-g-MOF-5	338	413

T_d: onset of decomposition temperature; T_{max}: maximum weight loss temperature.

Table S2. Mechanical properties of pristine Pebax and MMMs at various loadings of PIM-g-MOF.

Membrane	Elongation at Break (%)	Tensile Strength (MPa)	Young's Modulus (GPa)
Pebax	684.4	11.2	0.80
PIM-g-MOF-1	596.5	16.08	1.61
PIM-g-MOF-3	504.4	16.1	2.01
PIM-g-MOF-5	420.9	15.9	1.93

Table S3. Gas separation results of the MMMs

Membrane Code	[a] Permeability, Barrer			Selectivity, α	
	CO_2	N_2	CH_4	CO_2/N_2	CO_2/CH_4
Pebax	141.4	4.0	12.1	35.3	11.7
PIM-g-MOF-0.5	234	4.2	14.4	55.7	16.3
PIM-g-MOF-1	247	4.4	14.5	56.1	17
PIM-g-MOF-3	196.5	3.7	11.5	53.1	17.1
PIM-g-MOF-5	114.3	2.3	6.7	50	16.9

[a] Permeability in Barrer, where 1 Barrer $=10^{-10}$[cm^3 (STP) cm]/(cm^2 s cm Hg)

Table S4. Gas separation results of the pure MOF-containing mixed matrix membranes

Membrane Code	[a] Permeability, Barrer			Selectivity, α	
	CO_2	N_2	CH_4	CO_2/N_2	CO_2/CH_4
UiO-66-NH_2-1	150.2	5.4	11.2	27.8	13.4
UiO-66-NH_2-3	268.3	14.8	17.3	18.1	15.5

[a] Permeability in Barrer, where 1 Barrer $=10^{-10}$[cm^3 (STP) cm]/(cm^2 s cm Hg)
